# Notes and News

**Published:** 1902-12-13

**Authors:** 


### Notes and News.

Mr. H. Butlin, who has relinquished his post of surgeon at St. Bartholomew's Hospital, has been elected a consulting surgeon to the hospital.

Sir A. Conan Doyle's gift to the University of Edinburgh of £4,000, the result of the sale of his "History of the South African War," is to be applied to the foundation of a bursary of the value of £40 per annum to be awarded to the best South African student of each year without regard to nationality.

The Sanitary Board of Hong Kong, whilst insisting upon every house in the city being cleansed once a year, will no longer undertake the task of doing the cleansing itself. Locally this is regarded as a good measure, as the Chinese inhabitants are reported to be willing to do the work in their own houses, and the board will carry out an inspection to see that the cleansing is effective.

The new Ulster Medical Institute presented to the Ulster Medical Society by Professor Sir William Whitla has been opened by Lord Dudley. It is a handsome Gothic building, built of white glebe stone relieved by red Dumfries stone. In the library of the Institute are memorial windows to Dr. William Smyth and Dr. Brendon McCarthy, both of whom laboured in an heroic manner to overcome an epidemic of typhus on the island of Arranmore, in which Dr. Smyth lost his life.

The Special Christmas Supplement of The Hospital represents up-to-date word pictures of the position of the voluntary hospitals of London in the Coronation year. Included in it is a brief description of the work each one is doing, of its cost, and of its most pressing needs in the immediate future. The series of word pictures are presented under the title of "The Hospital Garden." We venture to think that the aspect of this garden will present features of special interest to our readers, and that within the limits of its enclosure is to be found all that is necessary to lead the way to a comprehension of the extensive field of hospital work.

A Roentgen Rays installation has been presented to the Cumberland Infirmary.

A medical history museum has been opened at Amsterdam. The museum forms part of the Communal Museum.

The municipal authorities of St. Petersburg have made arrangements for the disinfection of current silver coin.

Dr. Wyeth, of New York, has announced that he has found that an injection of water heated to from 190° to 212° Fahr. to be successful in the treatment of tumours.

The South-Eastern Railway Company has made arrangements for the issue of tickets at special rates to members of the International Congress of Medicine at Madrid who start from England.

Mr. C. Cochrane, of the Downs, St. Neots, announces that he will be glad to hear from persons interested in the subject of promoting the appointment of a Minister of Public Health and developing the Health Department of the Local Government Board into a separate Ministry.

The difficulty that medical men experience in diagnosing small-pox is shown by the fact that over 12 per cent. of mistaken diagnosis occurred amongst the patients sent to South Wharf for transmission to the Longreach hospitals. The disease most frequently taken for small-pox was chicken-pox, acne coming next in number.

The statistics of vaccination in New South Wales are startling. Only 2,081 vaccinations were performed, whilst the births numbered 37,826. Therefore the rate of vaccination was only 5½ per cent. Great care is exercised to prevent the introduction of small-pox into the country, but it can hardly be expected that one of the large towns will not one day experience an epidemic, and pay the great cost of cure, instead of the far less costly one of prevention.

Mr. George Herring has done a splendid work in maintaining the strong financial position of the Hospital Sunday Fund during the last three years. For three years he gave £10,000 per annum, and this year he did still more. Realising no doubt that in the Coronation year the Sunday Fund should show a better record than ever, and also that people had much to distract them, he had the courage to offer to give a sum equal to one quarter sum contributed by the congregations. His generous offer met with the result his intelligence had anticipated, and the Hospital Sunday Fund collection in the Coronation year was a record one. Mr. Herring in making a gift shows both a splendid generosity and keen intelligence. But Mr. Herring is a modest man, he does not practice the modern methods of self-advertisement, and so as far as general recognition is concerned he has yet to meet his deserts.

